# An S6:S18 complex inhibits translation of *E. coli rpsF*

**DOI:** 10.1261/rna.049544.115

**Published:** 2015-12

**Authors:** Arianne M. Babina, Mark W. Soo, Yang Fu, Michelle M. Meyer

**Affiliations:** Department of Biology, Boston College, Chestnut Hill, Massachusetts 02467, USA

**Keywords:** ribosome, gene regulation, translation inhibition, RNA *cis*-regulator, structured RNA

## Abstract

More than half of the ribosomal protein operons in *Escherichia coli* are regulated by structures within the mRNA transcripts that interact with specific ribosomal proteins to inhibit further protein expression. This regulation is accomplished using a variety of mechanisms and the RNA structures responsible for regulation are often not conserved across bacterial phyla. A widely conserved mRNA structure preceding the ribosomal protein operon containing *rpsF* and *rpsR* (encoding S6 and S18) was recently identified through comparative genomics. Examples of this RNA from both *E. coli* and *Bacillus subtilis* were shown to interact in vitro with an S6:S18 complex. In this work, we demonstrate that in *E. coli*, this RNA structure regulates gene expression in response to the S6:S18 complex. β-galactosidase activity from a *lacZ* reporter translationally fused to the 5′ UTR and first nine codons of *E. coli rpsF* is reduced fourfold by overexpression of a genomic fragment encoding both S6 and S18 but not by overexpression of either protein individually. Mutations to the mRNA structure, as well as to the RNA-binding site of S18 and the S6–S18 interaction surfaces of S6 and S18, are sufficient to derepress β-galactosidase activity, indicating that the S6:S18 complex is the biologically active effector. Measurement of transcript levels shows that although reporter levels do not change upon protein overexpression, levels of the native transcript are reduced fourfold, suggesting that the mRNA regulator prevents translation and this effect is amplified on the native transcript by other mechanisms.

## INTRODUCTION

Regulation of protein expression and activity occurs at many different stages between the DNA and the active protein product, starting with transcription initiation and ending with post-translational modifications. In bacteria, regulatory mechanisms affecting the synthesis, stability, or translational efficiency of the mRNA transcript are common, and RNA-based mechanisms are responsible for regulating a variety of processes. These include virulence (Gripenland et al. 2010; Sharma and Heidrich 2012) and stress response (Gottesman et al. 2006), as well as basic processes such as the biosynthetic pathways for essential metabolites (Sun et al. 2013) and ribosomal protein synthesis (Aseev and Boni 2011; Fu et al. 2013).

In *Escherichia coli*, ribosomes constitute >30% of all cellular proteins in actively growing cells (Harvey 1970). Furthermore, ribosome assembly is highly coordinated, and the stoichiometry of available components can significantly affect the efficiency and accuracy of this process (Sykes et al. 2010). Unsurprisingly, the synthesis of ribosomal proteins is also tightly regulated. The genes encoding ribosomal proteins are largely colocated in the genome to allow coordinated regulation of transcription initiation (Lemke et al. 2011). In addition, more than half of the ribosomal protein-encoding operons are further regulated by structured portions of their mRNA transcript (Zengel and Lindahl 1994; Fu et al. 2013). These RNA structures typically interact with one or more of the ribosomal proteins encoded by the transcript to inhibit gene expression. Although they are often found in the 5′ UTR of the transcript, these structures may also appear within intergenic regions (Mattheakis et al. 1989; Saito et al. 1994).

In *E. coli,* although several of the reported RNA structures have no characterized mechanism of action (Climie and Friesen 1988), most of those with known mechanisms appear to act by inhibition of translation initiation via ribosomal displacement or entrapment (Nomura et al. 1980; Draper 1987). This enables regulation of multiple ribosomal protein genes within an operon because many ribosomal protein genes display translational coupling, in which inhibition of translation for a gene at the 5′ end of a transcript also represses translation of the remaining downstream genes (Baughman and Nomura 1983; Thomas et al. 1987; Lindahl et al. 1989). In addition, inhibition of translation frequently leads to an increased degradation rate for the transcript, further repressing gene expression (Deana and Belasco 2005). Finally, regulation of Rho-independent transcription termination in response to ribosomal protein interactions also has been reported in both *E. coli* (Zengel and Lindahl 1990) and other bacterial species such as *Bacillus subtilis* (Choonee et al. 2007; Yakhnin et al. 2015).

Despite the importance of the RNA structures regulating ribosomal protein synthesis and their association and interactions with some of the most highly conserved proteins in bacteria, most of the structures observed in *E. coli* are not widely distributed to other phyla of bacteria (Fu et al. 2013). Instead it appears that alternative RNA regulators with distinct structures and mechanisms of action often accomplish analogous functions in different phyla of bacteria (Guillier et al. 2002; Choonee et al. 2007; Slinger et al. 2014). The diversity of RNA structures regulating ribosomal proteins follows the general trend that RNA regulators in bacteria are typically not widely conserved but evolve rapidly (Peer and Margalit 2011) and are present only in relatively narrow groups of bacteria (Lindgreen et al. 2014).

We and others recently reported a conserved RNA structure preceding *rpsF*, which encodes ribosomal protein S6 (Matelska et al. 2013; Fu et al. 2014). The RNA structure (rpsF_leader) is widely distributed to many bacterial species and displays some similarity with the S6:S18-binding site on the 16S rRNA. In many bacterial species, *rpsF* is colocalized in the genome with *priB*, which encodes a component of the primosome, and *rpsR*, which encodes ribosomal protein S18. In *E. coli*, this transcriptional unit also includes *rplI*, encoding ribosomal protein L9 (Isono and Kitakawa 1978). However, in many cases (including *B. subtilis*) *rplI* is found elsewhere in the genome. In the context of ribosome assembly, S6 and S18 form a heterodimer prior to interaction with the rRNA-S15 complex (Held et al. 1974; Agalarov and Williamson 2000; Recht and Williamson 2001). Examples of the conserved RNA structure preceding *rpsF* from *E. coli* and *B. subtilis* were found to specifically interact in vitro with an S6:S18 heterodimer with nM affinity (Matelska et al. 2013); however, the *B. subtilis* homolog also had weak, potentially nonspecific interactions with S18 in the absence of S6 (Fu et al. 2014). Additionally, similarities between the conserved RNA structure preceding *rpsF* and the rRNA-binding site of the S6:S18 heterodimer were identified. The combination of this in vitro binding data with the proximity of the RNA structure to the translation start codon strongly suggests that the conserved RNA structure allows regulation of these proteins in many species of bacteria.

In this work, we demonstrate that an example of the RNA structure from *E. coli* negatively regulates gene expression only in response to overexpression of both S6 and S18 using a *lacZ* reporter. This regulation may be disrupted by mutations to the regulatory RNA element that prevent its interaction with the repressor proteins, mutations to S18 that prevent interaction with the rRNA, and mutations to both S6 and S18 that prevent their interaction with one another. These results demonstrate that the S6:S18 complex is the biologically active effector. Furthermore, assessment of transcript levels by quantitative RT-PCR (qRT-PCR) shows that changes in *lacZ* mRNA levels do not correlate with the observed changes in β-galactosidase activity. Thus the mechanism by which this RNA regulates gene expression is most likely inhibition of translation. However, the native transcript is significantly decreased when S6 and S18 are both overexpressed, suggesting that inhibition of translation leads to either rapid decay of the transcript, or that S6 and S18 overexpression also inhibits transcription. Thus this mRNA structure joins a collection of mRNA structures in *E. coli* that together allow the fine-tuning of ribosomal protein levels across multiple transcriptional units.

## RESULTS AND DISCUSSION

### rpsF_leader is a regulatory element

**FIGURE 1.**
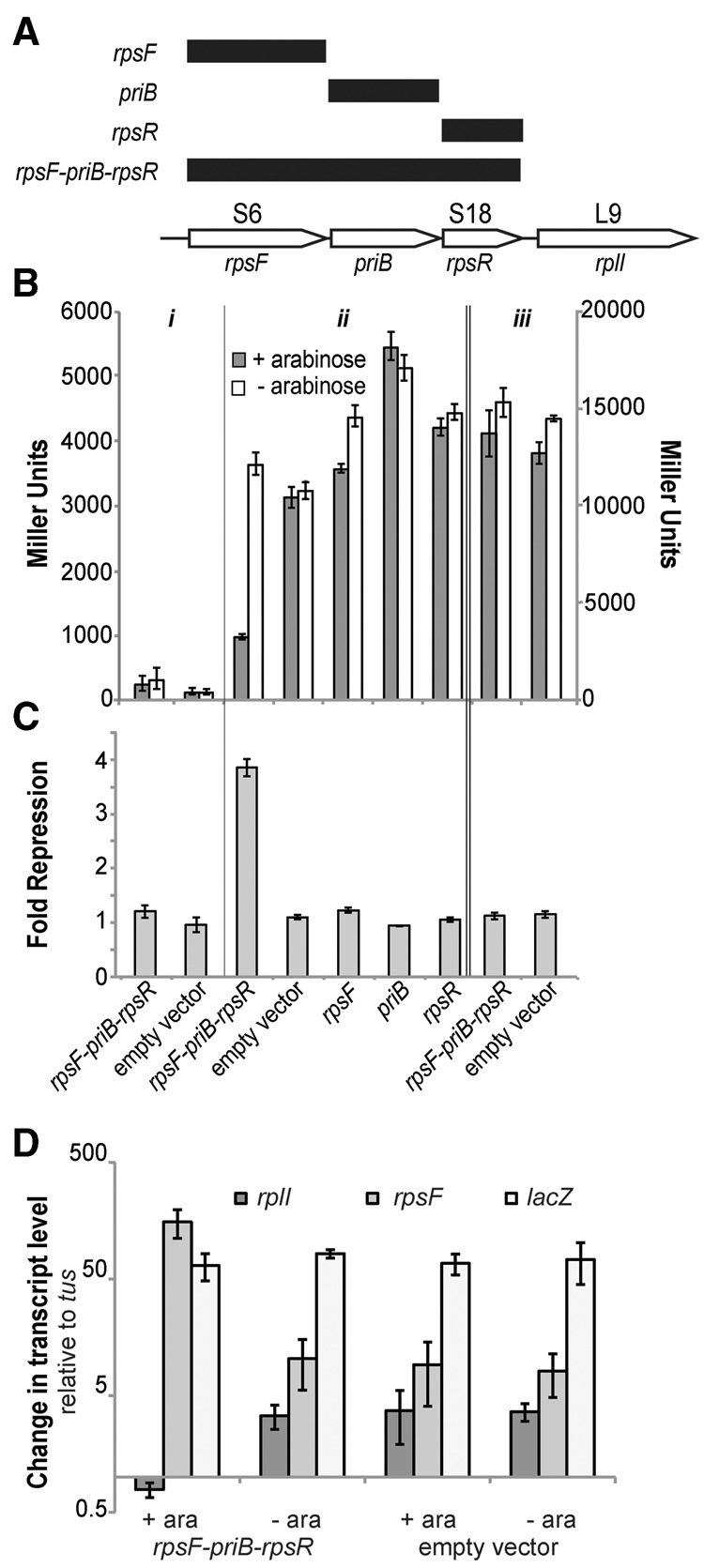
(*A*) Portions of the *rpsF* operon assessed in this study. (*B*) β-galactosidase activity of (i) cells with no *lacZ* reporter transcript induced (−IPTG); (ii) (*left* axis) cells with the *rpsF′-′lacZ* transcript induced (+IPTG) and different portions of an exogenous *rpsF-priB-rpsR* transcript (including an empty vector control) induced (+arabinose) and uninduced (−arabinose); and (iii) (*right* axis) cells with the *rpsO′-′lacZ* transcript induced (+IPTG) with the empty vector and the *rpsF-priB-rpsR* transcript, induced and uninduced (±arabinose). Error bars represent the standard error of the mean for biological replicates. (*C*) Fold repression of the *rpsF′-′lacZ* reporter construct derived from data in *B*. Fold repression is calculated from matched pairs of cultures as (β-galactosidase activity −arabinose)/(β-galactosidase activity +arabinose). Error bars represent standard error of the mean for this calculation for biological replicates. (*D*) qRT-PCR quantification of the native transcript, *rpsF-priB-rpsR-rplI* (*rplI*), overexpressed transcript (*rpsF*), and reporter transcript (*lacZ*) relative to the *tus* control transcript. Error bars represent standard error of the mean for biological replicates.

To assess the regulatory ability of the mRNA sequence preceding the *rpsF* gene in *E. coli* (rpsF_leader), we constructed a translational fusion between the RNA structure, including the first nine codons of *rpsF*, and *lacZ*, under transcriptional control of the Lac promoter (Slinger et al. 2014). To supply potential exogenous protein regulatory partners, we amplified portions of the *rpsF* operon and overexpressed them under the control of an arabinose inducible promoter on pBAD33 (Fig. 1A). These plasmids were cotransformed and the β-galactosidase activity of individual colonies was quantified with potential binding partners induced (+arabinose) and uninduced (−arabinose) (Fig. 1B). The assays were conducted with cells harvested during logarithmic phase growth (OD_600_ = 0.4–0.8) when the overexpressed protein-binding partners had been induced (+arabinose) for 2–3 h, and the reporter construct (*lacZ*) induced for 30 min. β-galactosidase activity of cells where the *lacZ* transcript is uninduced is negligible (Fig. 1B).

Using this system, overexpression of each individual component of the *rpsF* operon—*rpsF* encoding S6, *priB* encoding a component of the primosome, and *rpsR* encoding S18—results in little to no change in β-galactosidase activity (approximately onefold repression) compared to the empty vector control (pBAD33) (Fig. 1C). Upon expression of the first three genes of the *rpsF* operon (*rpsF-priB-rpsR*), we observe a large decrease in β-galactosidase activity (approximately fourfold repression). To control for potential global changes in translational efficiency that may be due to ribosome defects associated with overexpression of S6 and S18, we also examined the β-galactosidase activity of a similar *lacZ* fusion with an RNA structure not expected to interact with S6 or S18, that preceded ribosomal protein S15 in *E. coli* (*rpsO′-′lacZ*) (Philippe et al. 1993; Slinger et al. 2014). Although the *rpsO′-′lacZ* fusion results in higher β-galactosidase activity in comparison to the rpsF_leader-*lacZ* constructs (Fig. 1B), there is no significant change in β-galactosidase activity upon expression of the *rpsF-priB-rpsR* (S6:S18) construct compared to pBAD33 lacking any insert (Fig. 1C). Thus, overexpression of both S6 and S18 (*rpsF-priB-rpsR*) is necessary to regulate gene expression, and the observed change in gene expression is specific to the rpsF_leader.

### rpsF_leader inhibits translation

The *E. coli* example of the rpsF_leader contains a putative Shine–Dalgarno (SD) sequence within the structure, suggesting that the mechanism of action for this RNA is through inhibition of translation initiation. To examine the mechanism of regulation, we measured mRNA levels in our reporter strains via qRT-PCR to determine whether transcript levels are significantly altered under the conditions where we observe changes in reporter gene expression. The *tus* gene (terminus utilization substance) was used as a control transcript instead of a ribosomal protein or rRNA control transcript, as they might be affected by the overexpression of the *rpsF-priB-rpsR* fragment (Sykes et al. 2010). We measured levels of the overexpressed transcript (primers within *rpsF*), the reporter transcript (primers within *lacZ*), and the native transcript (primers within *rplI*) relative to *tus* transcript. We found that levels of the *lacZ* transcript do not change relative to the *tus* under the +arabinose condition where we observe changes in β-galactosidase activity (Fig. 1D). In contrast, while it is clear that the overexpression constructs increase *rpsF* transcript levels approximately 10-fold upon induction with arabinose as expected, the *rplI* transcript (corresponding to the native *rpsF-priB-rpsR-rplI* transcript) is reduced approximately 4.5-fold under these conditions. These results indicate that although the changes in β-galactosidase activity we observe are due to differences in translation, the native transcript is subject to additional regulation and is either not produced, terminates prematurely, or is degraded more rapidly under these conditions.

### Mutations to rpsF_leader affect expression and regulatory capacity

**FIGURE 2.**
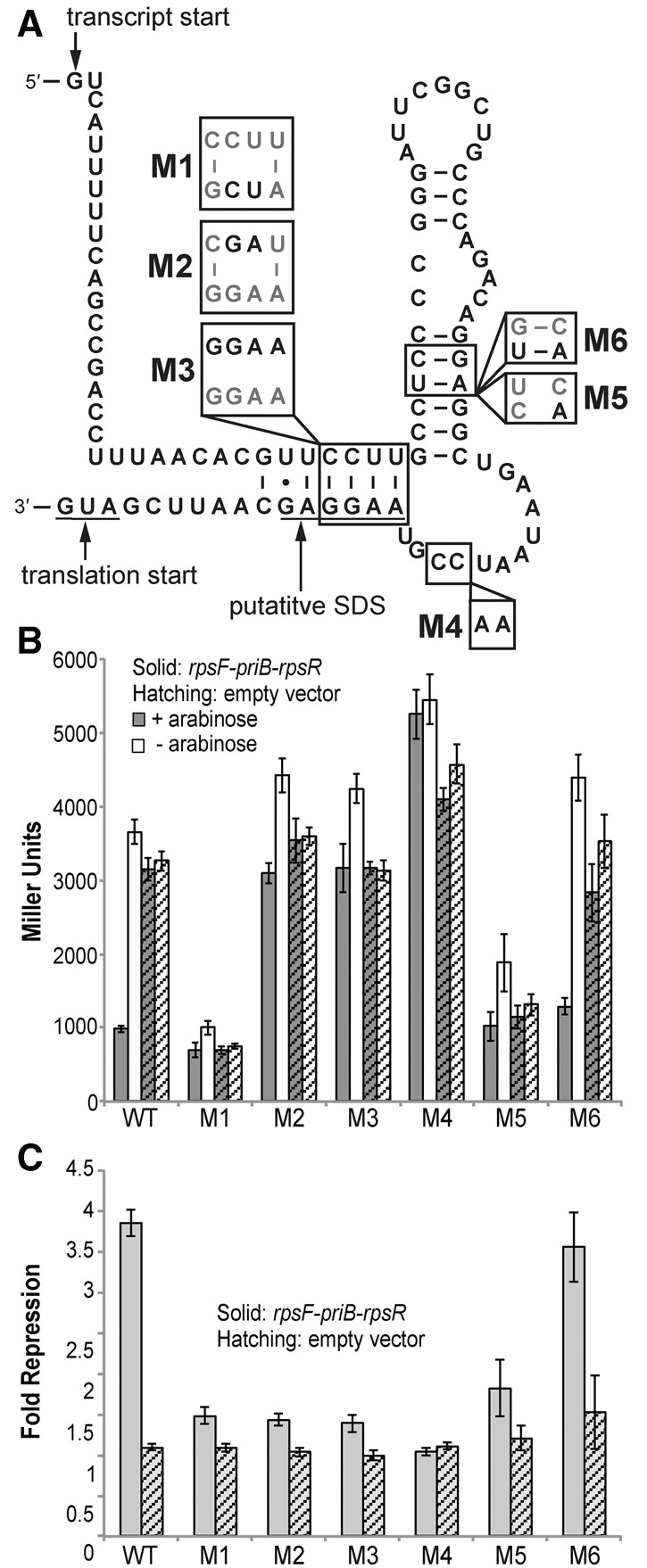
(*A*) The presumed secondary structure of the *rpsF* 5′ UTR used for reporter studies with mutations M1–M6. The transcription start site (Maciag et al. 2011), translational start, and putative Shine–Dalgarno (SD) sequence are indicated. (*B*) β-galactosidase activity of cells carrying plasmids with the unmutated rpsF_leader (WT) or each mutant RNA (M1–M6) and the *rpsF-priB-rpsR* overexpression plasmid or the empty vector (pBAD33) under induced and uninduced (±arabinose) conditions. (*C*) Fold repression as calculated in Figure 1C of the unmutated rpsF_leader (WT) and each mutant RNA (M1–M6).

To determine whether mutations to the RNA that prevent protein binding could abolish regulation, we examined six mutant RNAs (Fig. 2A). The M1 mutation disrupts the H1 stem as well as the putative SD sequence. This mutation in the homologous *B. subtilis* RNA was previously shown to reduce protein-binding affinity using in vitro electrophoretic mobility shift assays (EMSAs) (Fu et al. 2014). During our in vivo studies, we find that this mutation results in low β-galactosidase activity in both the presence and absence of exogenous protein (Fig. 2B). This indicates that this region is important for translational efficiency and supports our prediction that the H1 stem may contain the SD sequence. The overall low β-galactosidase activity of M1 makes any potential regulatory activity difficult or impossible to determine.

The M2 mutation is not directly in the proposed protein-binding region but is within positions that are predicted to pair with the SD sequence. This mutation in the homologous *B. subtilis* RNA completely abolished protein binding in vitro (Fu et al. 2014). Consistent with these data, we find that this mutation to the *E. coli* rpsF_leader almost completely abolishes regulation. Further disruption of this stem with the M3 mutation yields similar results, suggesting that the base-pairing in this region is important for protein binding and consequent regulation (Fig. 2C).

The M4 mutation is directly within the proposed protein-binding site, changing two highly conserved cytosines to adenines (Matelska et al. 2013; Fu et al. 2014). During in vitro studies of the homologous *B. subtilis* RNA, this mutation significantly inhibited protein binding. A more severe mutation at the same position (AAA rather than AAG) in the *E. coli* RNA had a similar effect during in vitro binding assays (Matelska et al. 2013). We find that this mutation completely abolishes the demonstrated regulation in vivo (Fig. 2C). This is consistent with its location directly within the protein-binding region.

The M5 mutation disrupts the highly conserved H2 stem of the rpsF_leader secondary structure. Mutations to this region in the homologous *B. subtilis* RNA significantly impaired RNA–protein interactions in vitro (Fu et al. 2014). In agreement with these data, this mutation reduces repression of β-galactosidase activity to about half of that observed with the wild-type rpsF_leader (∼1.8-fold repression). The low basal (unrepressed) β-galactosidase activity of the M5 mutant in comparison to the rpsF_leader wild type and other mutant constructs (Fig. 2B) does not appear to be due to changes in transcript levels (data not shown) but rather to translational efficiency. The M6 compensatory mutation to the H2 stem almost fully restores basal β-galactosidase activity and regulation to near-wild-type levels (∼3.5-fold repression) (Fig. 2B,C). This further confirms our previous secondary structure predictions and indicates that the base-pairing in the H2 stem is important for regulatory activity.

### Protein mutations prevent regulation

The S6:S18 complex is expected to be the biologically active regulator that interacts with the rpsF_leader. During ribosome assembly, S6 and S18 form a heterodimer prior to assembly with the rRNA-S15 complex (Held et al. 1974; Recht and Williamson 2001). The rpsF_leader shows significant sequence and structural similarities with the S18-binding site of the rRNA, and tertiary structure modeling indicates that the majority of RNA–protein contacts are likely with S18 (Matelska et al. 2013). In vitro studies of the *B. subtilis* rpsF_leader homolog show weak and likely nonspecific interactions between S18 and the rpsF_leader (Fu et al. 2014), and our β-galactosidase assay results (Fig. 1B,C) indicate that overexpression of both S6 and S18 is required for inhibition.

**FIGURE 3.**
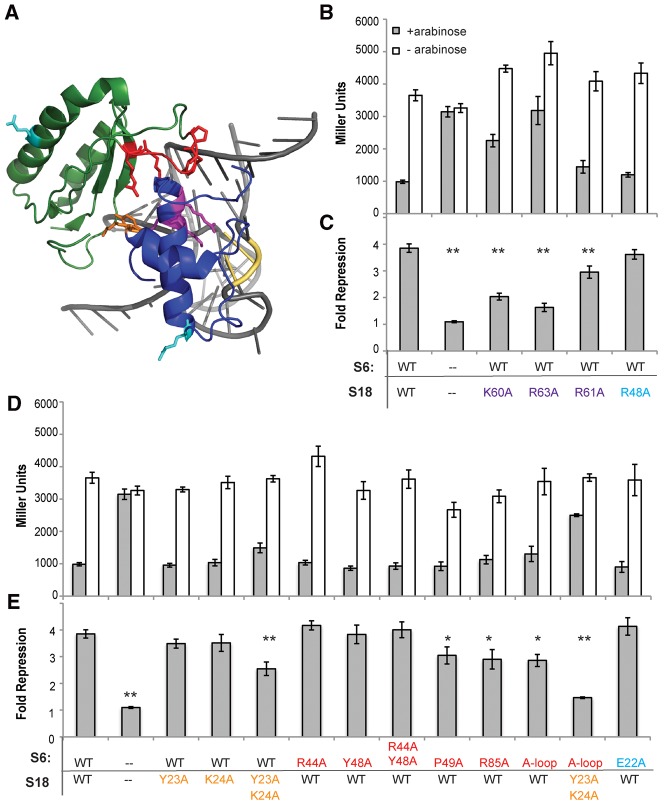
(*A*) Rendering of S6:S18 heterodimer in complex with the rRNA (coordinates derived from 2QAL [Borovinskaya et al. 2007]). The rRNA segment (660–678; 713–739) is gray, interacting bases C719 and C720 are highlighted in yellow, S18 is shown in blue, amino acids mutated at the S18:RNA interface (K60, R61, and R63) are indicated in purple, and amino acids mutated at the S6:S18 interface (Y23 and K24) are orange. S6 is displayed in green, and the amino acids mutated in the S6:S18 interface are highlighted in red. For individual amino acids mutated at the S6:S18 interface (R44, Y48, P49, and R85), side chains are displayed; for the additional amino acids altered in the “A-loop” mutant (44–49 all mutated to alanine), only the backbone is colored. Negative control mutations (S6 E22 and S18 R48) are highlighted in cyan. (*B*) β-galactosidase activity of cells carrying the *rpsF-priB-rpsR* overexpression construct with mutations to the S18 RNA-binding region with protein induced and uninduced (±arabinose). For comparison, data for the unmutated construct and empty vector (--) are included. (*C*) Fold repression for S18 RNA-binding site mutations calculated as described in Figure 1C. (**) Statistically significant change (*P* < 0.01) from the wild-type construct. (*D*) β-galactosidase activity of cells carrying the *rpsF-priB-rpsR* overexpression construct with mutations made to the S6:S18 interface. For comparison, data for the unmutated construct and empty vector (--) are included. (*E*) Fold repression for S18:S6 binding interface mutations calculated as described in Figure 1C. (**) *P* < 0.01, (*) *P* < 0.05.

To assess the role S18 has in the mRNA–protein interactions, we altered two positively charged amino acids in S18 (within the *rpsF-priB-rpsR* construct) that are expected to interact with the conserved pair of cytosines in both the rRNA and rpsF_leader (lysine 60 and arginine 63 to alanine) (Fig. 3A). These amino acids form hydrogen bonds with the conserved cytosines in the loop region adjacent to helix 23a of the rRNA (C719 and C720) (Agalarov et al. 2000) that are mimicked in the mRNA structure by the conserved pair of cytosines mutated in M4 (Fig. 2A). During in vitro studies, mutating these amino acids resulted in either significant reduction in binding affinity (K60A) or complete loss of saturated binding (R63A) (Matelska et al. 2013). In our assays, both of these mutants significantly reduce regulation (approximately twofold repression, *P* < 0.01) (Fig. 3B,C). Additionally, mutating arginine 61 to an alanine, a residue that is implicated in both S18-rRNA interactions and S6:S18 dimer interactions in a *Thermus thermophilus* structure of the S15:S6:S18:rRNA complex (Agalarov et al. 2000) but only appears to contact the RNA in crystal structures of the *E. coli* ribosome (Borovinskaya et al. 2007), results in a modest decrease in regulatory activity (∼2.9-fold repression, *P* < 0.01) (Agalarov et al. 2000). This supports the conclusion that S18 contacts with the mRNA are critical for binding and subsequent regulatory activity. Arginine 48 was also mutated to an alanine as a negative control. R48 is not known to directly contribute to S18-rRNA or S6:S18 protein–protein interactions, and this mutation to S18 did not affect regulation.

To further assess whether the S6:S18 heterodimer is the biologically relevant effector, we mutated residues in both S6 and S18 (within the *rpsF-priB-rpsR* construct) to disrupt S6:S18 dimer interactions and consequent regulation. However, the interface of S6 and S18 proved difficult to completely disrupt using mutations to a single protein. Mutations to S18, tyrosine 23, and lysine 24 to alanine individually had little or no effect on regulation. However, combining these mutations results in a modest, but significant (*P* < 0.01) decrease in β-galactosidase activity (2.5-fold repression). On the complementary S6 surface, individual and combined mutations of S6 arginine 44 and tyrosine 48 to alanine had little effect on regulation (Fig. 3C). Mutations of proline 49 and arginine 85 to alanine and replacing residues 44–49 (RQLAYP) with alanine (“A-loop”) all had modest, but significant effects on repression (decreasing to approximately threefold, *P* < 0.05). However, combining the S6 “A-loop” mutation with the S18 Y23A/K24A mutation did strongly impact regulation (1.5-fold repression, *P*≪0.001), indicating that disruption of the S6:S18 interface can disrupt regulation. A negative control mutation to S6, glutamate 22 to alanine, showed no change in repression.

We suspect the robustness of the protein–protein interaction is due to several factors. First, our overexpressed mutant proteins are competing with endogenous protein levels, and we may see less of an effect due to this competition. Second, the nature of the S6:S18 protein–protein interaction may be somewhat plastic and robust to our mutagenesis efforts. The structures of the *T. thermophilus* S15:S6:S18:rRNA complex and *E. coli* full ribosomes are not identical, leading to slightly varying determinations of which residues are most critical for this interaction (Agalarov et al. 2000; Borovinskaya et al. 2007). In particular the N-terminal portion of S18 that contacts S6 is not resolved in the *T. thermophilus* complex, suggesting that there may be some flexibility in the S6:S18 interaction. That our combined S6 “A-loop” and S18 Y23A/K24A mutations had the strongest impact on regulation indicates that the first of these two factors is likely playing a significant role, and supports our conclusion that the active complex is the S6:S18 heterodimer.

### Concluding remarks

In this work, we show that in *E. coli*, the rpsF_leader RNA is a regulatory element that inhibits the translation of *rpsF*. Changes in β-galactosidase activity are not accompanied by corresponding changes in the level of *lacZ* transcript. However, the native transcript does show significant reduction under conditions where β-galactosidase activity is reduced, suggesting that it is subject to additional regulation. Because of past associations of reduced translation with more rapid transcript degradation (Deana and Belasco 2005), we strongly suspect this mechanism may play a significant role in amplifying the relatively modest fourfold repression we observe.

Our mutagenesis studies indicate that the biologically relevant effector is the S6:S18 heterodimer. Unlike many regulators of ribosomal protein synthesis, the rpsF_leader does not respond to a primary rRNA-binding protein but rather a complex of secondary rRNA-binding proteins. This situation is relatively rare, as most characterized regulators to date interact with primary rRNA-binding proteins, although there are a few exceptions (e.g., S2, L25; Aseev et al. 2008, 2015). The closest comparison to this situation is the *rplJ-rplL* regulator that interacts with either L10 or the L10(L12)_4_ complex (Yates et al. 1981).

The rpsF_leader is one of the few RNA structures responsible for ribosomal protein regulation that is widely distributed across many bacterial phyla (Matelska et al. 2013; Fu et al. 2014). For other such widely distributed regulators, the same protein-binding site may be utilized for different mechanisms of gene regulation. For example, in *E. coli* the L10(L12)_4_ regulatory mRNA enables transcription inhibition (Johnsen et al. 1982; Christensen et al. 1984), whereas in *B. subtilis* the conserved L10(L12)_4_ mRNA-binding site regulates transcription attenuation (Iben and Draper 2008; Yakhnin et al. 2015). This is similar to many riboswitch aptamers where the same recognizable aptamer may utilize different expression platforms in different species (Barrick and Breaker 2007). Therefore, it would not be unlikely for the *rpsF*_leader to utilize distinct mechanisms for regulation in diverse species of bacteria. Confirmation of the biological activity of the rpsF_leader mRNA structure allows it to join a still growing canon of RNA regulatory structures in bacteria that allow regulation of ribosomal protein synthesis but utilize a wide variety of mechanisms and have very different distribution patterns across bacterial phyla.

## MATERIALS AND METHODS

### Plasmids

The *lacZ* reporter plasmid is a modified version of the pLac-ThiM#2-tetA-gfpuv plasmid previously described (Muranaka et al. 2009; Slinger et al. 2014), in which the tetA-gfpuv construct is replaced with a *lacZ* gene using SalI and XbaI restriction sites. The fragment corresponding to the RNA and the first nine codons of *rpsF* (NC_000913.3/4425023-4425144) was PCR amplified from genomic DNA extracted from *E. coli* strain XL1-Blue (Agilent Technologies) and cloned between the EcoRI and SalI sites, to generate an RNA-*lacZ* translational fusion. To construct pBAD33 (ATCC 87402) overexpression plasmids, genomic fragments corresponding to portions of the *rpsF* operon (NC_000913.3 *rpsF*: 4425118-4425513; *priB*: 4425520-4425834; *rpsR*: 4425839-4426066; *rpsF-rpsR*: 4425118-4426066) were PCR amplified from genomic DNA from *E. coli* and cloned between the XbaI and SacI sites*.* To enable efficient translation, a Shine–Dalgarno sequence (5′-AGGAGGTTTTAAA) was appended to the 5′ end of each genomic fragment. Mutant RNA and protein plasmids were created by site-directed mutagenesis from the original plasmids. All plasmid sequences were confirmed by Sanger sequencing.

### β-galactosidase activity assays

To generate strains for β-galactosidase activity analysis, a reporter plasmid and a protein overexpression plasmid were cotransformed into *E. coli* strain NCM534 [F−, Δ*(araD-araB)714*, Δ*(lacA-lacZ)880(::FRT)*, *lacIp-4000(lacI^Q^)*, *zah-2225::FRT*, λ^−^, Δ*araEp-532::FRT*,φ*P*_*cp18*_*araE533*, *rph-1*] (*E. coli* Genetic Stock Center, strain #: 8256). For each independent assay, a single colony was chosen and grown overnight at 37°C with shaking (225 rpm) in 1.5 mL LB +ampicillin (100 µg/mL) +chloramphenicol (35 µg/mL). This culture was used to inoculate two separate prewarmed 1.5 mL cultures, one containing 15 mM l-arabinose (+arabinose) to induce protein overexpression and one without any sugar added (−arabinose, protein not induced), to a starting OD_600_ of ∼0.05–0.1. Cultures were grown at 37°C with shaking (225 rpm) until an OD_600_ of ∼0.4–0.6 was reached. IPTG (1 mM) was added to both cultures to induce transcription of the reporter transcript. The induced cultures were incubated for 30 min at 37°C with shaking (225 rpm). Spectinomycin (100 µg/mL) was added to each culture following the 30-min incubation to inhibit further translation. Cells (1 mL) were harvested and resuspended in 1 mL Z buffer (50 mM Na_2_HPO_4_, 40 mM NaH_2_PO_4_, 10 mM KCl, 1 mM MgSO_4_, and 50 mM 2-mercaptoethanol) + 100 µg/mL spectinomycin. OD_600_ readings were recorded as cell suspensions in Z buffer. Samples that had an OD_600_ reading of 0.4 or lower at time of harvest were discarded. β-galactosidase assays were performed as previously described using 30 µL of cell suspensions and Miller units were calculated as follows (Miller 1992):

$$\text{Miller Units}=1000\times \frac{A_{420}}{\Delta t\;(\text{min})\times A_{600}\times \text{vol}\;(\mathrm{mL})}.$$

To determine the fold repression for each sample, the Miller units of the −arabinose (protein not induced) culture were divided by that of the corresponding +arabinose (protein induced) culture. The values reported represent five or more independent replicates for wild-type, mutant rpsF-leader, and protein overexpression cotransformed strains, and three independent replicates for the uninduced (−IPTG) and *rpsO′-′lacZ* control assays. Error bars represent standard error on the mean across biological replicates. To determine significance, the fold repression of samples was compared using a Welch's *t*-test.

### Quantitative RT-PCR

Total RNA was extracted using TRIzol (Life Technologies) from +arabinose and −arabinose cultures grown essentially as described. Contaminating DNA was removed from 10 µg of total RNA by RQ1 DNase digestion at 37°C for 4 h (Promega) followed by phenol/chloroform extraction and ethanol precipitation. Reverse transcription reactions were conducted with random hexamers using SuperScript III (Life Technologies) on 2.5 µg of DNAse-treated RNA according to the manufacturer's instructions. The resulting cDNA was used as template for qPCR using an Applied Biosystems 7500 qPCR machine (SYBR Green detection, Thermo Scientific). qPCR primers targeting the *lacZ* reporter (5′-TACCTGTTCCGTCATAGCGA, 5′-CTGTTTACCTTGTGGAGCGA), the overexpression construct (*rpsF* 5′-GGCTTACCCGATCAACAAAC, 5′-CGGAAGGTAGTTTCCAGCTC), and the native transcript (*rplI* 5′-TACCATCGCGTCTAAAGCTG, 5′-TTCGCTCTTAGCCACTTCAA) were used to quantify transcript levels in each sample. Quantification of *tus* was used as a normalization control (5′-TGTTTTCGAAGCGACAGATG, 5′-TTTCGAGGCCGAGAATTTTA). Equivalent experiments were conducted on a reaction lacking reverse transcriptase to ensure that DNAse digestion removed all contaminating DNA. Each condition is represented by three independent biological replicates, and qPCR was conducted with three technical replicates for each biological replicate. Standard error reported represents variance among biological replicates.
